# Rapid identification of *Burkholderia mallei* and *Burkholderia pseudomallei* by intact cell Matrix-assisted Laser Desorption/Ionisation mass spectrometric typing

**DOI:** 10.1186/1471-2180-12-229

**Published:** 2012-10-10

**Authors:** Axel Karger, Rüdiger Stock, Mario Ziller, Mandy C Elschner, Barbara Bettin, Falk Melzer, Thomas Maier, Markus Kostrzewa, Holger C Scholz, Heinrich Neubauer, Herbert Tomaso

**Affiliations:** 1 Friedrich-Loeffler-Institut, Federal Research Institute for Animal Health, Institute of Molecular Biology, Südufer 10, Greifswald-Insel Riems, D-17493, Germany; 2 Bundeswehr Institute of Microbiology, Neuherbergstrasse 11, Munich, D-80937, Germany; 3 Working Group Biomathematics, Friedrich-Loeffler-Institut, Federal Research Institute for Animal Health, Südufer 10, Greifswald-Insel Riems, D-17493, Germany; 4 Friedrich-Loeffler-Institut, Federal Research Institute for Animal Health, Institute of Bacterial Infections and Zoonoses, Naumburger Str. 96 a, Jena, 07743, Germany; 5 Bruker Daltonik GmbH, Fahrenheitstr. 4, Bremen, 28359, Germany

## Abstract

**Background:**

*Burkholderia (B.) pseudomallei* and *B. mallei* are genetically closely related species. *B. pseudomallei* causes melioidosis in humans and animals, whereas *B. mallei* is the causative agent of glanders in equines and rarely also in humans. Both agents have been classified by the CDC as priority category B biological agents. Rapid identification is crucial, because both agents are intrinsically resistant to many antibiotics. Matrix-assisted laser desorption/ionisation mass spectrometry (MALDI-TOF MS) has the potential of rapid and reliable identification of pathogens, but is limited by the availability of a database containing validated reference spectra. The aim of this study was to evaluate the use of MALDI-TOF MS for the rapid and reliable identification and differentiation of *B. pseudomallei* and *B. mallei* and to build up a reliable reference database for both organisms.

**Results:**

A collection of ten *B. pseudomallei* and seventeen *B. mallei* strains was used to generate a library of reference spectra. Samples of both species could be identified by MALDI-TOF MS, if a dedicated subset of the reference spectra library was used. In comparison with samples representing *B. mallei*, higher genetic diversity among *B. pseudomallei* was reflected in the higher average Eucledian distances between the mass spectra and a broader range of identification score values obtained with commercial software for the identification of microorganisms. The type strain of *B. pseudomallei* (ATCC 23343) was isolated decades ago and is outstanding in the spectrum-based dendrograms probably due to massive methylations as indicated by two intensive series of mass increments of 14 Da specifically and reproducibly found in the spectra of this strain.

**Conclusions:**

Handling of pathogens under BSL 3 conditions is dangerous and cumbersome but can be minimized by inactivation of bacteria with ethanol, subsequent protein extraction under BSL 1 conditions and MALDI-TOF MS analysis being faster than nucleic amplification methods. Our spectra demonstrated a higher homogeneity in *B. mallei* than in *B. pseudomallei* isolates. As expected for closely related species, the identification process with MALDI Biotyper software (Bruker Daltonik GmbH, Bremen, Germany) requires the careful selection of spectra from reference strains. When a dedicated reference set is used and spectra of high quality are acquired, it is possible to distinguish both species unambiguously. The need for a careful curation of reference spectra databases is stressed.

## Background

*Burkholderia (B.) pseudomallei* and *B. mallei* are genetically closely related bacterial species that can cause fatal disease in humans and animals. *B. pseudomallei* is a facultative intracellular soil bacterium and the cause of melioidosis, which has the highest prevalence in the hot and humid regions of Southeast Asia, and Northern Australia. The infection can be acquired by contact with contaminated soil or water by inhalation or percutaneously. Human infections can be asymptomatic, can present with focal lesions that may affect almost any organ, or may be disseminate resulting in pneumonia and septicaemia. In certain regions of Asia melioidosis is a major cause of human morbidity and acute systemic melioidosis has a case fatality rate of up to 50% even if treated [1,2]. Melioidosis has been described in wild animals, but also in farm and pet animals and can be spread by animal trade and transport [3]. Both species are pathovars of a single genomospecies which was divided historically in two separate species due to their clinical impact and host tropism. *B. thailandensis* is the third closely related species of the so-called “Pseudomallei complex” which has been out-grouped from the species *B. pseudomallei* based on arabinose fermentation and its markedly lower pathogenicity. *B. thailandensis* and *B. pseudomallei* are soil bacteria that share the same geographical distribution.

*B. mallei* is a gram-negative, non-motile obligate pathogen and the causative agent of glanders and farcy in equines (horses, donkeys, mules). In horses, glanders primarily presents with purulent nasal discharge, inflammation of the mucous membranes of the upper respiratory tract, and poor general condition, whereas farcy is a chronic cutaneous disease with formation of nodules which may develop into ulcers. Equines are the only known reservoir. Contact with infected animals, ingestion of glanderous meat and exposure to aerosols can cause *B. mallei* infections in humans. Human glanders is highly lethal and resembles melioidosis. Chronic and latent infections can exacerbate into the acute form even after 15 years in both diseases. Both bacterial species are intrinsically resistant to many antibiotics including ampicillin and broad- and expanded-spectrum cephalosporines due to the production of a beta-lactamase [4]. *B. mallei* and *B. pseudomallei* have been classified by the CDC as priority category B biological agents.

Isolation and microbiological identification of *B. pseudomallei* and *B. mallei* from clinical samples can take up to one week. Commercial biochemical test systems for *B. mallei* are not available and *B. pseudomallei* may be misidentified as *Chromobacterium violaceum* or other bacteria [5-7]. Latex agglutination using a monoclonal antibody was shown to be a valuable technique for the rapid identification of *B. pseudomallei* in positive blood cultures, but no commercial tests are available [8,9]. Real-time PCR systems have been developed for diagnosing and differentiating as rapid alternatives to biochemical tests, but few have been validated on clinical samples [10-13].

Matrix-assisted laser desorption ionization-time of flight mass spectrometry (MALDI-TOF MS) for identification of bacteria has become a useful tool for the rapid identification of bacteria (see [14] for a recent review). In some studies intact cell mass spectrometry (ICMS) showed better correlation to genetic markers than conventional morphological classification [15]. The technique and modified procedures including a digestion step (‘shotgun mass mapping’) have been successfully used for subspecies-level classification in some species [16]. Characteristic features of ICMS are simple sample preparation procedures of whole cells, spectrum acquisition in the mass range between approximately 2,000 and 15,000 Da and analysis based upon comparison of sample spectra with reference spectra. By statistical approaches, similarity between mass spectra can be exploited for the identification of microorganisms. MALDI-TOF MS was also established for identification of non-fermenting gram-negative bacteria isolated from cystic fibrosis patients in Brazil [17]. Patients with cystic fibrosis suffer primarily under infections with Pseudomonads, but *Burkholderiae* play also an important role. In the Brazilian study a comprehensive number of *Burkholderia* species was included and could be identified correctly in most cases. However, neither *B. pseudomallei* nor *B. mallei* were among the clinical isolates tested. Sporadic cases of melioidosis in cystic fibrosis patients have been described in the literature and seem to be an emerging problem [18-22]. Due to increased travel activity, international trade, climate change, and the potential threat of bioterrorist attacks infections caused by *B. pseudomallei* and *B. mallei* can become a serious problem.

The aim of this study was to evaluate the potential benefit of MALDI-TOF MS for the rapid and reliable identification and differentiation of *B. pseudomallei* and *B. mallei*.

## Results

### Construction of a reference database

**Figure 1:**
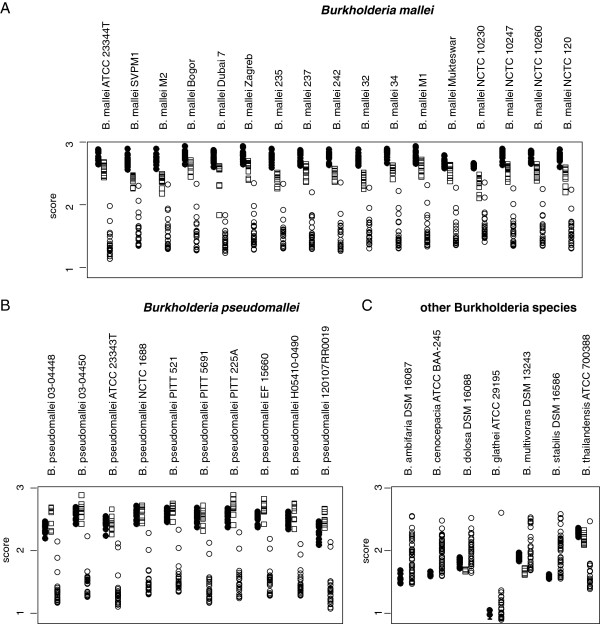
**Summary of the MALDI Biotyper queries with the reference spectrum set.** The three panels summarize the score-oriented hit lists that the thirty-four strains of the custom reference set produced when queried against the reference spectrum set plus all representatives of the *Burkholderia* genus present in the MALDI Biotyper reference database. The three panels represent queries of *B. mallei* (**A**), *B. pseudomallei* (**B**) and other members of the B. genus (**C**). Filled circles, squares and open circles indicate scores produced by database entries representing *B. mallei, B. pseudomallei* or any of the other species in the reference database. Note that for all samples the highest ranking hit represents a member of the respective *Burkholderia* species.

A custom made set of 34 reference spectra, which are called main spectra (MSP) in the MALDI Biotyper terminology (Bruker Daltonik GmbH, Bremen, Germany), was generated and used as the basis for all further calculations. This reference spectra set included all strains listed in Table 1 (*B. mallei* and *B. pseudomallei*) and additionally samples from *B. ambifaria* (DSM 16087), *B. cenocepacia* (ATCC BAA-245), *B. dolosa* (DSM 16088), *B. glathei* (ATCC 29195), *B. multivorans* (DSM 13243), *B. stabilis* (DSM 16586), and *B. thailandensis* (ATCC 700388). This set of 34 samples will be referred to as the ‘custom reference set’. The full set of MALDI Biotyper reference database entries will be referred to as ‘MALDI Biotyper reference set’. In a first analysis, spectra of the custom reference set were queried against a combined database composed of the custom reference set of 34 *Burkholderia* samples and the MALDI Biotyper reference set. For every queried spectrum, MALDI Biotyper software generates a score-based ranked list of organisms. The organism with the highest score is ranked first (‘top hit’) and its species is taken as the result of the query. MSP scores are calculated by an algorithm that compares the masses of a tested sample with those present in the MSP of the spectra collection by agglomeration of scores for every mass and performing a normalisation that will result in a final value between 0 (unrelated) and 1000 (identical). For convenience, the result is given as logarithmic value smaller than or equal to 3.0. Masses of the tested spectrum will be scored in a weighted fashion depending on their location within a narrower or a wider mass tolerance window centred on the masses of the MSP. Additionally, the score for every coinciding mass of the tested spectrum will be weighted according to the frequency with which the corresponding mass of the MSP has been found in the single spectra that were used for the construction of the MSP. Thus, scores carry information on the number of coinciding masses found in the tested spectrum and the MSP, the mass aberration that is observed between the corresponding masses of the tested spectrum and the MSP and the reproducibility of the respective masses of the MSP. Cut-off values for reliable species determination cannot be theoretically calculated and have to be determined empirically. According to the manufacturer, experience has shown that scores exceeding 2.0 will allow reliable genus identification and species identification in the majority of cases. Scores calculated for all spectra of the custom reference set among them are summarized in Figure 1. In the hit lists of all tested specimen, the highest-ranking entry represented the same species as the tested specimen, indicating that, within the given database, the standard MALDI Biotyper identification procedure reliably allows the determination of *Burkholderia* species including the differentiation between *B. mallei* and *B. pseudomallei*. Even though species identification was correct in all cases, the distribution of scores in Figure 1 gave rise to concern about the reliability of the discrimination of the three members of the Pseudomallei group: *B. thailandensis* produced relatively high scores with some of the *B. mallei* and *B. pseudomallei* samples, and *B. pseudomallei* generally produced relatively high scores with *B. mallei*. Therefore, a set of *B. mallei* and *B. pseudomallei* samples was additionally cultivated and processed in two different laboratories and queried using the custom reference set as database in order to challenge the identification procedure. It is known that cultivation conditions can influence the outcome of ICMS experiments. In an interlaboratory comparison that was performed in three laboratories with *B. thailandensis* we had observed that cultivation on different growth media (Columbia 5% Sheep Blood agar (CSB), chocolate agar, and McConkey agar) and different cultivation periods (24, 48 and 72 h) had a notable influence on the scores in the identification procedure (data not shown). To avoid any variance caused by differing growth conditions, all *B. mallei* and *B.pseudomallei* were grown on CSB and the cultivation period of 48 h was strictly observed.

**Table 1:** ***Burkholderia *(B.) *mallei *and *B. pseudomallei *strains**

**Bacteria**	**Origin**	**Country**	**Year**	**fliC**	**fliP**	**Motility**
*B. mallei*						
ATCC 23344^T^	Human	China	1942	+	+	-
NCTC 120	unknown	United Kingdom	1920	+	+	-
NCTC 10230	Horse	Hungary	1961	+	+	-
NCTC 10247*	Human	Turkey	1960	+	+	-
NCTC 10260	Human	Turkey	1949	+	+	-
M1*	unknown	unknown	unknown	+	+	-
M2	unknown	unknown	unknown	+	+	-
Rotz7 (SVP)	unknown	unknown	unknown	+	+	-
32	unknown	unknown	unknown	+	+	-
34	unknown	unknown	unknown	+	+	-
235	unknown	unknown	unknown	+	+	-
237	unknown	unknown	unknown	+	+	-
242	unknown	unknown	unknown	+	+	-
Bogor	Horse	Indonesia	unknown	+	+	-
Mukteswar	Horse	India	unknown	+	+	-
Zagreb	Horse	former-Yugoslavia	unknown	+	+	-
Dubai 7	Horse	United Arab Emirates	2004	+	+	-
*B. pseudomallei*						
ATCC 23343^T^	Human	unknown	<1957	+	-	+
EF 15660*	unknown	unknown	unknown	+	-	+
NCTC 1688*	Rat	Malaysia	1923	+	-	+
PITT 225A*	Human	Thailand	1986	+	-	+
PITT 521	Human	Pakistan	1988	+	-	+
PITT 5691	unknown	unknown	unknown	+	-	+
120107RR0019	Human	Italy	2007	+	-	+
H05410-0490	Human	Asia	unknown	+	-	+
03-04448	Human	unknown	unknown	+	-	+
03-04450	unknown	unknown	unknown	+	-	+

^T^ type strain.

*Constituents of the reduced reference set dedicated for the discrimination of *B. mallei* and *B. pseudomallei*.

Characteristics of *Burkholderia (B.) mallei* and *B. pseudomallei* strains used to establish the database for the identification and differentiation with MALDI-TOF mass spectrometry. Species identity was confirmed by real-time PCR assays targeting a sequence of the *fliC* gene that is specific for both species but does not discriminate *B. mallei* from *B. pseudomallei*. The real-time PCR assay targeting *fliP* is specific for *B. mallei*. Motility was also assessed as a phenotypic marker because *B. pseudomallei* is motile while *B. mallei* is not.

### Discrimination of *B. mallei* and *B. pseudomallei*

Scores between *B. mallei* samples listed in Table 1 ranged between 2.56 and 2.94, whereas those between *B. pseudomallei* samples ranged between 2.25 and 2.89. For *B. mallei* samples, the score range over 2.72 was completely reserved for correct species assignments and the top scores of all isolates reached this threshold. Due to the stronger variation of *B. pseudomallei*, such a well-defined threshold for correct species assignments could not be defined for this species.

**Figure 2:**
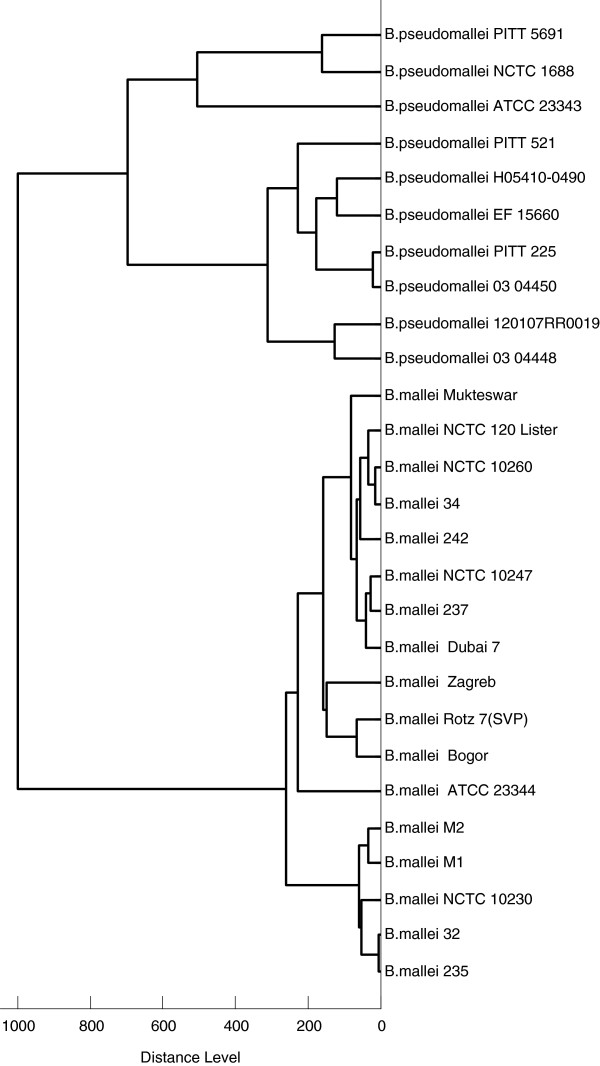
**Dendrogram obtained for *Burkholderia mallei *and *Burkholderia pseudomallei *strains.** Spectrum-based distances between members of the *B. mallei* species are usually smaller than between representatives of *B. pseudomallei.*

**Figure 3:**
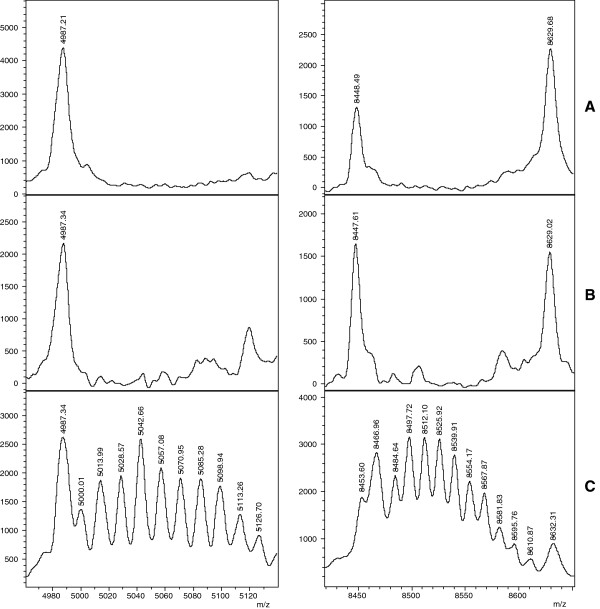
**Unique modification patterns found for two proteins of *B. pseudomallei *ATCC23343**^**T**^**.** Two regions of representative spectra of the three strains *Burkholderia(B.) mallei* Bogor (panel **A**), *B. pseudomallei* NCTC 1688 (panel **B**) and *B. pseudomallei* ATCC 23343 (panel **C**) are shown. Two striking series of multiple peaks with m/z distances of 14 Da were observed in *B. pseudomallei* ATCC 23343 but in no other of the tested isolates.

**Figure 4:**
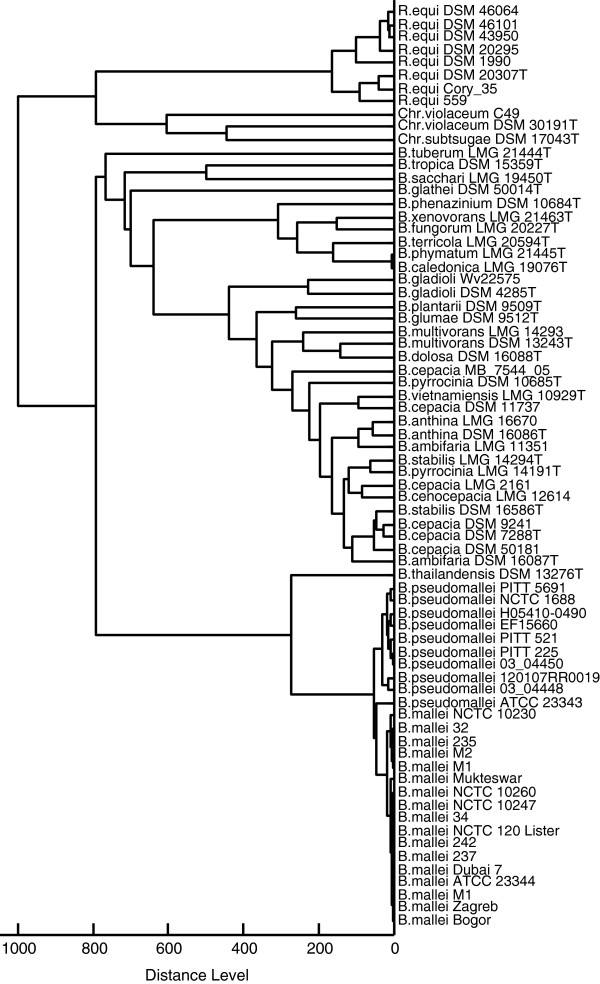
**Spectrum-based dendrogram representing *Burkholderia mallei, Burkholderia pseudomallei, *and other relevant bacteria.** The dendrogram was constructed based on the MALDI Biotyper scores. Note that distances between *B. mallei* and *B. pseudomallei* isolates are small compared to the distances of other B. species. *B. mallei/B. pseudomallei* and *B. thailandensis* separate as distinct group from the other species of the B. genus.

As MSP will usually not be constructed for routine samples, the identification procedure with MALDI Biotyper was repeated with single spectra of the *B. mallei* and *B. pseudomallei* samples from Table 1. The results were very similar to those obtained with MSP. For *B. mallei* samples, scores between 2.60 and 2.93 were observed, whereas *B. pseudomallei* were recognized with scores in the range from 2.57 to 2.92. The top-ranking hit of the hit-list correctly indicated the species of all queried samples. Scores of all top-ranking hits exceeded 2.8. Construction of a score-based dendrogram of *B. mallei* and *B. pseudomallei* samples (Figure 2) with MALDI Biotyper software resulted in the expected clustering of the two species. Interestingly, the *B. pseudomallei* type strain ATCC 23343 separated notably from other *B. pseudomallei* representatives. This was at least in part caused by the appearance of two series of masses between 5,000 and 5,084 Da and 8,500 and 8,565 Da which were not detected in any of the other samples (Figure 3). The observation of multiple mass differences of 14 Da in these series suggests that they were caused by multiple methylations being specific for this strain. The mass series reproducibly appeared in all single spectra used to calculate the MSP of the *B. pseudomallei* strain ATCC 23343 and were also observed in independent replicates of the spectra with a freshly cultivated specimen. The identity of the modified molecule is unknown. A dendrogram was constructed from the MSP of the *B. mallei* and *B. pseudomallei* strains listed in Table 1 and the *Burkholderia, Chromobacterium,* and *Rhodococcus* species from Table 2 which were added from the MALDI Biotyper database (Figure 4). As expected, score-based distances between *B. mallei* and *B. pseudomallei* were smaller than between the other *Burkholderia* species and *B. mallei/B. pseudomallei* and *B. thailandensis* formed a distinct group which was separated from the other species of the *Burkholderia* genus.

**Table 2:** Bacteria investigated for specificity testing

**Species**	**Strain**
*Burkholderia (B.) ambifaria*	LMG 11351
*B. ambifaria*	DSM 16087 ^T^
*B. anthina*	DSM 16086 ^T^
*B. anthina*	LMG 16670
*B. caledonica*	LMG 19076 ^T^
B. caribensis*	DSM 13236 ^T^
*B. cenocepacia*	LMG 12614
B. cenocepathia*	ATCC BAA-245
*B. cepacia*	MB_7544_05
*B. cepacia*	DSM 11737
*B. cepacia*	18875_1 CHB
*B. cepacia*	DSM 9241
*B. cepacia*	DSM 50181
*B. cepacia*	LMG 2161
B. cepacia*	DSM 7288 ^T^
*B. cepacia*	ATCC 25416 ^T^
*B. dolosa*	DSM 16088
*B. fungorum*	LMG 20227 ^T^
*B. gladioli*	Wv22575 CHB
*B. gladioli*	DSM 4285 ^T^
*B. glathei*	DSM 50014 ^T^
*B. glumae*	DSM 9512 ^T^
*B. multivorans*	LMG 14293
*B. multivorans*	DSM 13243 ^T^
*B. phenazinium*	DSM 10684 ^T^
*B. phymatum*	LMG 21445 ^T^
*B. plantarii*	DSM 9509 ^T^
*B. pyrrocinia*	DSM 10685 ^T^
*B. pyrrocinia*	LMG 14191 ^T^
*B. sacchari*	LMG 19450 ^T^
*B. stabilis*	LMG 14294 ^T^
*B. stabilis*	DSM 16586 ^T^
*B. terricola*	LMG 20594 ^T^
*B. thailandensis*	DSM 13276 ^T^
B. thailandensis*	ATCC 700388
*B. tropica*	DSM 15359 ^T^
*B. tuberum*	LMG 21444 ^T^
*B. vietnamiensis*	LMG 10929 ^T^
*B. xenovorans*	LMG 21463 ^T^
*Chromobacterium (C.) subtsugae*	DSM 17043 ^T^
*C. violaceum*	C49 MVO
*C. violaceum*	DSM 30191^T^
*Rhodococcus (R.) equi*	DSM 1990
*R. equi*	DSM 20295
*R. equi*	DSM 20307 ^T^
*R. equi*	DSM 43950
R. equi*	DSM 44426
*R. equi*	DSM 46064
*R. equi*	559 LAL

^T^ type strain.

List of bacteria to be differentiated from *Burkholderia mallei and Burkholderia pseudomallei* using MALDI-TOF mass spectrometry. These bacteria include closely related bacteria, possible sample contaminants, bacteria with very similar clinical presentation and other relevant bacteria. MSP reference spectra were constructed for the species indicated with an asterisk (*); all other samples indicate isolates of the MALDI Biotyper database.

**Figure 5:**
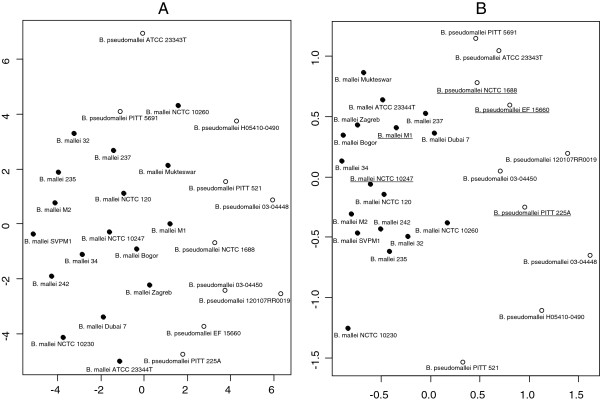
**Sammon representation of the spectrum-based distance relations of *B. mallei *and *B. pseudomallei. ***Diagrams **A** and **B** were calculated from qualitative or quantitative distance matrices derived from the mass alignment of the spectra, respectively. Members of the dedicated reference spectrum set for the discrimination of *B. mallei* and *B. pseudomallei* are underlined. Sammon representations allow visualising distance matrices in a two-dimensional plot with minimized distortion.

The distance relations of *B. mallei* and *B. pseudomallei* were further analysed after transfer of the mass lists into statistical programming language R. Based on the mass alignment, a cluster analysis was performed, a distance matrix was calculated, and the distances within and between the *B. mallei* and *B. pseudomallei* strains were calculated. To test the influence of the peak intensities on the clustering behavior, cluster analysis was performed with the quantitative and qualitative data. For the latter purpose the quantitative alignment containing the intensities of every mass peak was transformed into a qualitative binary table by marking the absence or presence of a mass with 0 and 1, respectively. From both tables, distance matrices were calculated and visualized as Sammon-plots (Figure 5). For qualitative and quantitative data the average normalized distances between *B. mallei* strains were smaller than between *B. pseudomallei* strains (0.57 vs. 0.73 for the binary data and 0.46 vs. 0.71 when peak intensities of the spectra were included) confirming the score-based clustering in Figure 2 that suggests a higher variation among *B. pseudomallei* than among *B. mallei* strains. As a measure for the separation of the two species, the weighted ratio between the distances of *B. mallei* and *B. pseudomallei* strains within the species and the distances between the species was calculated. Transition from qualitative to quantitative data showed slight improvement (0.82 vs. 0.74) in the species separation indicating that peak intensities are relevant for the discrimination of the two species and should not be neglected. Cluster analysis with the quantitative data using the k-means algorithm indicated the presence of two clusters which were congruent with the two *Burkholderia* species whereas cluster analysis based on the qualitative data failed to do so. On basis of the qualitative data, which weights every mass equally for the calculation of the distance, *B. pseudomallei* ATCC 23343 was notably separated from all other spectra, most probably because of the multiple modifications shown in Figure 3.

As some *B. mallei* and *B. pseudomallei* specimen from the reference spectrum set produced quite high scores with the respective other species, it was essential to test the practicability of the custom reference set in a routine laboratory setting with samples prepared in a different laboratory. The panel of samples used for this test (Table 3, the ‘test set’) only partially overlapped with the custom reference set (Table 1) so that not only inter-laboratory variation was tested but also the ability of the custom reference set to discriminate newly appearing isolates like those from a glanders outbreak in the United Arabic Emirates in 2004.

**Table 3:** **Bacteria used to test the reliability of ICMS-based discrimination of *Burkholderia mallei *and *Burkholderia pseudomallei***

**Species**	**Strain designation**	**Score**
*B. mallei*	32	2.470
	34	2.475
	237	2.189
	242	2.550
	ATCC 23344^T^	2.382
	Bogor	2.522
	Mukteswar	2.554
	Zagreb	2.472
	NCTC 120	2.478
	NCTC 10260	2.092
	NCTC 10247	2.325
	NCTC 10230	1.960
	05-767	2.329
	05-762	2.515
	05-2316	2.496
	Dubai3-10, 14-17*	2.437 - 2.630
*B. pseudomallei*	EF 15660	2.692
	NCTC 1688	2.489
	06-2372	2.588
	06-2377	2.621
	06-2379	2.427
	06-2388	2.603
	06-2393	2.328
	06-2395	2.633
	06-772	2.379

*B. mallei isolates from several horses isolated during the glanders outbreak in UAE 2004.

List of strains used to evaluate the reliability of ICMS-based discrimination of *B. mallei* and *B. pseudomallei* using a dedicated set of reference strains. Column ‘Score’ designates the score value of the top-ranking hit in the dedicated database, which in all cases represented the same species as the tested sample. (^T^, typestrain).

Using the complete custom reference set as database a number of misclassifications of the test set isolates occurred. Considering the distribution of scores (Figure 1) and the distance relations between *B. mallei* and *B. pseudomallei* (Figure 5), this was not unexpected and obviously a consequence of the indiscriminate inclusion of all available *B. mallei* and *B. pseudomallei* samples into the custom reference set. Classification could be substantially improved by selecting combinations of isolates of *B. mallei* and *B. pseudomallei* to form a dedicated reference set which is optimized for the discrimination of the two species. To screen the complete custom reference set of *B. mallei* and *B. pseudomallei* for appropriate combinations of isolates, the outcome of a database query was simulated with all permutations of up to four members of each species. The smallest reference group yielding error-free results was composed of two *B. mallei* (M1, NCTC10247) and three *B. pseudomallei* (EF15660, PITT 225A, NCTC01688) isolates which are highlighted by an asterisk in Table 1. Not surprisingly, these isolates located close to the centers of their respective species in the Sammon plot visualization of the distance matrix (Figure 5).

**Figure 6:**
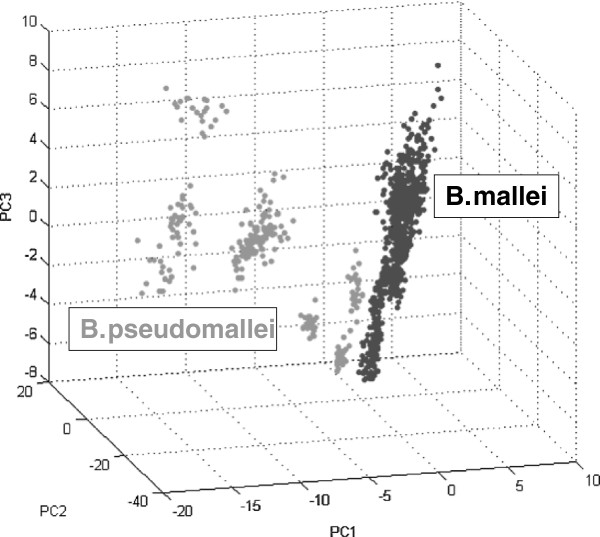
**Principal component analysis of spectra derived from *B. mallei *and *B. pseudomallei. ***Principle Component Analysis of ten strains of *B. mallei* and ten strains of *B. pseudomallei*, respectively. The unsupervised statistical analysis separates both species based on the three major principle components. While *B. mallei* form a relatively uniform cluster, significant diversity can be observed for *B. pseudomallei*. Analysis of the spectra from the specimens in Table 1 yielded very similar results (data not shown).

Finally, multivariate statistics on basis of the four different statistical approaches (Genetic Algorithm, Support Vector Machine, Supervised Neural Network, Quick Classifier) available in ClinProTools 3.0 showed that *B. mallei* and *B. pseudomallei* could be well separated with cross validation results ranging between 98.95% and 100.00% (data not shown). Principal Component Analysis (PCA) carried out with ClinProTools 3.0 (Figure 6) further confirmed the separation of both species and also the broader distribution of *B. pseudomallei* in comparison with *B. mallei*.

### Identification of taxon-specific biomarker ions

Mass spectra of the reference spectrum set were analysed for species-specific masses which may be used for species identification independent of the score values considered so far. For that purpose the mass lists of the MSP generated with MALDI Biotyper software were evaluated in detail. An alignment of all masses occurring in the spectra was constructed as a table in which every column represented the mass spectrum of a sample and every row the intensity of a mass occurring in a certain mass range. The alignment contained a total of 350 masses. One of them, 4,410 Da, was found in the spectra of all 34 samples and also in every one of the single spectra underlying the MSPs so that this mass can be considered a reliable indicator of the set of nine *Burkholderia* species that was analysed (*B. mallei, B pseudomallei, B, thailandensis, B. ambifaria, B. cenocepacia, B. dolosa, B. glathe, B. multivorans, B. stabilis*). Seven more masses (3,655 [doubly charged 7,309], 5,195, 6,551, 7,169, 7,309, 8,628 and 9,713 Da) were present in all *B. mallei* and *B. pseudomallei* samples but also in one or more of the other *Burkholderia* species. Considering the close relation of *B. thailandensis* with *B. mallei* and *B. pseudomallei*, mass 9,713 Da is of interest, which was specific for all *B. mallei, B. pseudomallei,* and *B. thailandensis* samples, i.e. the Pseudomallei group. Finally, 6,551 Da was present in all *B. mallei* and *B. pseudomallei* samples but in none of the other species, making it an effective discriminator between the *B. mallei/pseudomallei* group and the other representatives of the genus *Burkholderia*. Concerning the distinction of *B. mallei* and *B. pseudomallei*, statistical analysis with ClinProTools 3.0 software revealed a number of masses with significant class separation between the two species based on peak intensity. Most significant separation could be obtained based on the masses 7,553 and 5,794 which differ significantly in intensity between the two species.

## Discussion

In recent years MALDI-TOF MS has been introduced in microbiological laboratories as a time saving diagnostic approach supplementing morphological, biochemical, and molecular techniques for identification of microbes [23]. In several studies the comparability with conventional identification procedures was assessed with generally good correlation, but discordances were seen on the species and even on the genus level [24,25]. This proteomic profiling approach was successfully applied in routine identification of bacterial isolates from blood culture with the exception of polymicrobial samples and streptococci [26]. The identification of *Burkholderia* spp. and other non-fermenting bacteria using MALDI-TOF MS was investigated in cystic fibrosis (CF) patients as *Burkholderia* spp. (mainly of the cepacia-complex) cause a relevant number of life-threatening infections in these patients [27-29]. It was demonstrated that MALDI-TOF MS is a useful tool for rapid identification in the routine laboratory. *B. pseudomallei* can be the cause of melioidosis in CF patients and travelers to tropical regions, but this bacterium and the closely related species *B. mallei* was not included in previous MALDI-TOF MS studies [18-22,30,31]. Natural catastrophes like the tsunami in Indonesia (2004) and occasional flooding in other tropical regions resulted in elevated incidence of melioidosis and several cases among travelers and tourists [32-36]. *B. mallei* and *B. pseudomallei* are biological agents which further underlines the need for rapid detection tools. Identification of *Burkholderia* ssp. and distinction of *B. mallei* and *B. pseudomallei* from other species was feasible. This was also true for the closely related bacterium *B. thailandensis*. All strains grew well within 48 hours and could then be readily prepared for MALDI-TOF MS.

Due to the close relationship of *B. mallei* and *B. pseudomallei*, it was not surprising that the search for species-identifying biomarker ions discriminating these species was not successful. Obviously, more complicated mass signatures are required for this purpose and, as we could show after separate statistical evaluation of qualitative and quantitative data, peak intensities also play a crucial role for the discrimination of *B. mallei* and *B. pseudomallei*. However, group-specific masses like 9,713 Da, standing for the Pseudomallei complex (*B. mallei/B. pseudomallei/B. thailandensis)* or 6,551, exclusively found in *B. mallei* and *B. pseudomallei* may be of use for the discrimination of these three species.

For the identification of *B. mallei* and *B. pseudomallei* samples under routine laboratory conditions, it was necessary to reduce the reference spectrum set to avoid misclassifications. Interestingly, the reference spectrum set optimized for spectrum-based discrimination neither contained the type strain ATCC 23344^T^ (*B. mallei*) nor ATCC 23343^T^ (*B. pseudomallei*). One reason for the exclusion of ATCC 23343 could be the occurrence of two peak series with repeating mass increments of 14 Da most probably representing polymethylated proteins. This strain has been shown to have unique immunological features. In an immunization experiment with a panel of 14 *B. pseudomallei* strains, ATCC 23343 induced monoclonal antibodies in mice which did not cross-react with any of the other *B. pseudomallei* strains [37]. These peculiarities may indicate that this type strain has been genetically modified by frequent subcultivation or misuse of media. To our knowledge, similar modifications which may have an impact on classification of bacteria have not been reported to-date. These series were specific for the isolate and also for two molecules within the observed mass range.

## Conclusions

In this study we have demonstrated that isolates of the closely related species *B. mallei* and *B. pseudomallei* can be identified using MALDI-TOF MS. Dangerous and cumbersome handling under BSL 3 conditions can be minimized by inactivation of the isolates with ethanol and subsequent MALDI-TOF MS analysis that requires much less time than nucleic acid amplification methods [38]. The reference spectra exhibited a higher homogeneity among *B. mallei* than among *B. pseudomallei*. The type strain of *B. pseudomallei* ATCC 23343 was isolated decades ago and separated from the other *B. pseudomallei* specimens in the dendrograms which is probably due to polymethylation as indicated by two intensive series of mass increments of 14 Da. To our knowledge, this is the first report of such a modification in whole cell MALDI-TOF MS spectra of microorganisms. As expected for closely related species, especially when one of them, *B. pseudomallei*, displays the broad distribution of MALDI-phenotypes that was observed, the identification process requires the selection of spectra from representatives of the centers of their respective distance distributions. Uncritical inclusion of all available samples as references in a library was counterproductive for the identification process. Only by selecting an appropriate set of reference spectra (Table 3) it was possible to identify all strains. This underlines the need for careful curation of reference spectra databases used for the identification of microorganisms.

## Methods

### Bacterial strains

A comprehensive collection of *B. mallei* and *B. pseudomallei* strains, referred to as the ‘reference set’, were tested (Table 1) and compared with spectra of closely related and other clinically relevant bacteria (Table 2) included in the MALDI Biotyper Reference Library (version 3.0, Bruker Daltonics, Bremen, Germany). Strain identity was confirmed using Gram staining, motility testing, and real-time PCR assays targeting *fliC* and *fliP* as described previously [11,12], and a species-specific DNA-microarray [39]. Strains were obtained from the Friedrich-Loeffler-Institut, Jena, Germany and the Bundeswehr Institute of Microbiology in Munich, Germany. Strain Dubai 7 was kindly provided by the Central Veterinary Reference Laboratory, Dubai, UAE. Some strains originated from the Robert Koch Institute in Berlin, Germany, that coordinated a project of the European Union for the “Establishment of Quality Assurances for Detection of Highly Pathogenic Bacteria of Potential Bioterrorism Risk”. Spectra from the set of strains enlisted in Table 3, referred to as ‘test set’, were recorded with an Autoflex mass spectrometer (Bruker) in a second laboratory and queried against the reference set to test for robustness and inter-laboratory variation.

### Intact cell mass spectrometry (ICMS)

Samples were prepared as described previously [16,40]. Briefly, the bacteria were cultivated under BSL 3 conditions on a nutrient blood agar containing 3% glycerol at 37°C for 48 hours. Specimens from single colonies were thoroughly suspended in 300 μl water and precipitated by addition of 900 μL ethanol (98% v/v). This treatment inactivated the bacteria as was demonstrated by growth controls and the specimens could be further tested under BSL 1 conditions. After sedimentation for five minutes at 10,000 g min^-1^ the supernatant was carefully removed and the sediment suspended in 50 μL of 70% (v/v) formic acid. After mixing with 50 μL acetonitrile, the suspension was centrifuged as described above and the supernatant transferred to a fresh tube. 1.5 μL of the extract was spotted onto a steel MALDI target plate and allowed to dry at ambient temperature. Finally, the dried extract was overlaid with 2 μL of a saturated solution of α-Cyano-4-hydroxycinnamic acid in 50% acetonitrile/2.5% trifluoroacetic acid as matrix and was again allowed to dry. A custom-made database of reference spectra was generated using the MALDI Biotyper software (version 2.0, Bruker Daltonik GmbH, Bremen, Germany) following the guidelines of the manufacturer. Each sample was spotted onto six target spots of a steel MALDI target plate. Spectra were acquired with an Ultraflex^TM^ I instrument (Bruker Daltonik GmbH) in the linear positive mode in the range of 2,000 to 20,000 Da. Acceleration Voltage was 25 kV and the instrument was calibrated in the range between 3,637.8 and 16,952.3 Da with Bacterial Test Standard calibrant (BTS, Bruker Daltonik GmbH). Four single mass spectra with 500 shots each were acquired from each spot and a reference spectrum calculated from the 24 single spectra. Reference spectra contained the parameters peak mass and intensity and additional information on the reproducibility of the mass peaks, i.e. the frequency of occurrence of every peak in the underlying 24 single spectra. Reference spectra were generated within the mass range of 2,000 to 20,000 Da with the default parameter settings in the MALDI Biotyper software. The number of peaks was limited to 100 per reference spectrum and all peaks of a reference spectrum were normalized to the most intense peak with an intensity of 1.0. The minimum frequency of occurrence within the 24 single spectra was set to 50% for every mass. Peaklists of reference spectra were exported for further evaluation in the statistical programming language R.

To test the inter-laboratory variation and the robustness of the classification by using MALDI Biotyper software, a set of *B. mallei* and *B. pseudomallei* test samples from a second laboratory (Table 3) was queried against the reference spectra set described above. These spectra were recorded at the Bundeswehr Institute of Microbiology with an Autoflex mass spectrometer (Bruker Daltonik GmbH, Bremens). Spectra were generated for the test set in the same way as for the reference set. A query of all test samples was performed and the resulting scores were transferred into a data matrix, the ‘score matrix’, in which every column represented a member of the reference set and every line a test sample. The power of certain combinations of representatives of the two classes within the reference set to discriminate samples of the test set was tested as follows: the columns representing the combination of reference spectra to be evaluated were selected from the score matrix and every member of the test set was classified by assigning it to the class of the member of the reduced reference set with the highest score. The number of correct and incorrect assignments was then calculated for the test set. This procedure simulates a MALDI Biotyper query with a reduced number of spectra in the reference database.

### Transfer of data into statistical programming language R

Peak lists of the reference spectra generated by the MALDI Biotyper software were converted into a format compatible with the R-package caMassClass which offers a number of functions to evaluate mass spectra [41] for further statistical analysis in R version 2.10.1. available at the R-project homepage [42]. Peak lists were aligned by the msc.peaks.align command of caMassClass and transformed into a binary mass table where rows represented all unique masses of the aligned spectra set and every column represented the spectrum of one sample. The size of the mass ranges defining a unique peak in the alignment, designated as bin size, was restricted to a maximum of 2,000 ppm. Among other features, the algorithm of the msc.peaks.align command minimizes the bin size in the given range, maximizes the space between bins and ensures that no two peaks of the same spectrum are in the same bin. For the calculation of qualitative data, the presence of the respective mass in the spectrum of a sample was marked with 1, absence with 0, i.e. all mass intensities were removed. These tables were the basis for the calculation of distances (R-routine ‘dist’, parameter ‘binary’ for the distance measure) which were used for the construction of cladograms, Sammon plots [43], and k-means cluster analysis using the R-routines ‘hclust’ (parameter ‘ward’ for the agglomeration method) [44], ‘sammon’ (used with default settings) and ‘kmeans’ (three initial cluster centers, maximum of 100 iterations, Hartigan-Wong algorithm [45]).

### Statistical analysis with ClinProTools software

Raw spectra from the specimens in Table 3 were imported into ClinProTools 3.0 software for statistical analysis. Each species was represented by 20 to 24 spectra to cover measurement variability. The multiple spectra of multiple species were imported as a “class” for the respective species. ClinProTools preformed a normalization and recalibration of mass spectra before further analysis, thereby reducing measurement variability effects significantly. Peak picking was performed based on the overall average spectrum over the whole mass range (signal to noise threshold of 5). Further spectra processing parameters were: baseline correction (convex hull), resolution (300 ppm), smoothing (Savitzky Golay, 5 cycles with 2 m/z width),

Multivariate statistical analyses were performed using the four supervised algorithms and PCA which are implemented in ClinProTools. For the Genetic Algorithm, models with maximum 5 peaks and 50 generations were calculated and k-nearest neighbor (kNN) classification was performed with 5 neighbors. Also for Support Vector Machine the maximum number of peaks was set to 5 and kNN classification was performed with 5 neighbors. Supervised Neural Network was calculated with automated optimization of peak number, maximum 25. For the Quick Classifier, a maximum number of differentiating peaks of 25 was allowed; selection of peaks was based on ranking in t-test. For PCA, “level” scaling was selected.

## Authors’ contributions

AK performed MALDI-TOF MS experiments, data analysis and participated in drafting the manuscript. RS worked in the BSL3 laboratory, performed MALDI-TOF MS experiments and data analysis. MZ developed R-scripts and participated in the mathematical analysis of mass spectra and in solving classification problems. MCE coordinated the work in the BSL3 laboratory, performed cultivation and PCR assays. BB performed MALDI-TOF MS experiments and data analysis. FM worked in the BSL3 laboratory, performed cultivation and PCR assays. TM performed MALDI-TOF MS experiments and data analysis. MK performed data analysis and statistical examination. HCS worked in the BSL3 laboratory, performed cultivation and PCR assays, and critically reviewed the manuscript. HN critically reviewed the manuscript. HT participated in the design of the study, coordinated the experiments, and participated in drafting the manuscript. MK and TM are employees of Bruker Daltonik GmbH, the manufacturer of the MALDI Biotyper system used in this study. All authors read and approved the final manuscript.
